# Acceptability and feasibility of home-based hypertension and physical activity screening by community health workers in an under-resourced community in South Africa

**DOI:** 10.1007/s10389-023-01873-w

**Published:** 2023-03-23

**Authors:** Mark Stoutenberg, Simone H. Crouch, Lia K. McNulty, Andrea Kolkenbeck-Ruh, Georgia Torres, Philippe J. L. Gradidge, Andy Ly, Lisa J. Ware

**Affiliations:** 1 Department of Kinesiology, College of Public Health, Temple University, 237 Pearson Hall, 1800 North Broad, Philadelphia, Pennsylvania 19122 USA; 2 Centre for Exercise Science and Sports Medicine, School of Therapeutic Sciences, Faculty of Health Sciences, University of the Witwatersrand, Wits Education Campus, 27 St. Andrews Road, Parktown, Gauteng, 2193 South Africa; 3 SA MRC/Wits Developmental Pathways for Health Research Unit (DPHRU), School of Clinical Medicine, Faculty of Health Sciences, University of the Witwatersrand, Corner College and Clinic Road, Chris Hani Baragwanath Academic Hospital, Soweto, 1864 South Africa; 4 Cardiovascular Pathophysiology and Genomics Research Unit, School of Physiology, Faculty of Health Sciences, University of the Witwatersrand, 7 York Road, Johannesburg, 2193 South Africa; 5 DSI-NRF Centre of Excellence in Human Development, University of the Witwatersrand, Wits Education Campus, 27 St. Andrews Road, Parktown, Gauteng, 2193 South Africa

**Keywords:** Community health worker, Home visit, Hypertension, Non-communicable disease, South Africa

## Abstract

**Background:**

Low–middle-income countries (LMICs) face increasing burdens from non-communicable disease (NCDs) requiring primary care task shifting to community health workers (CHWs). This study explored community members' perceptions of NCD-focused, CHW-led home visits in a historically disadvantaged township of South Africa.

**Methods:**

Trained CHWs visited community member homes, performing blood pressure and physical activity (PA) screenings, followed by brief counselling and a satisfaction survey. Semi-structured interviews were conducted within 3 days of the visit to learn about their experiences.

**Results:**

CHWs visited 173 households, with 153 adult community members consenting to participate (88.4%). Participants reported that it was easy to understand CHW-delivered information (97%), their questions were answered well (100%), and they would request home service again (93%). Twenty-eight follow-up interviews revealed four main themes: 1) acceptance of CHW visits, 2) openness to counselling, 3) satisfaction with screening and a basic understanding of the results, and 4) receptiveness to the PA advice.

**Conclusion:**

Community members viewed CHW-led home visits as an acceptable and feasible method for providing NCD-focused healthcare services in an under-resourced community. Expanding primary care reach through CHWs offers more accessible and individualized care, reducing barriers for individuals in under-resourced communities to access support for NCD risk reduction.

## Background

The burden of non-communicable diseases (NCDs) in sub-Saharan Africa has increased in recent years (Gouda et al. 2019). Primary hypertension, defined in South Africa as a resting systolic blood pressure ≥ 140 mmHg or diastolic blood pressure ≥ 90 mmHg and/or use of antihypertensive medication to control blood pressure (Rayner et al. 2019), is a leading cause of cardiovascular-associated mortality (Olsen et al. 2016; Zhou et al. 2021). The prevalence of hypertension has increased significantly in the last 30 years due to poor detection and blood pressure control and management, with low- and middle-income countries (LMICs) experiencing the highest burden (NCD Risk Factor Collaboration 2021). Several modifiable risk factors, including obesity, poor quality diets with excess sodium consumption, and physical inactivity, contribute to the development of hypertension (Mirmiran et al. 2018; Masilela et al. 2020). While knowledge about hypertension and associated behavioural risk factors is increasing, there is still a need for efforts to increase health awareness (Jongen et al. 2019).

Community health workers (CHWs) have become a critical part of healthcare service infrastructure in many countries (LeBan et al. 2021). CHWs provide additional workforce where resources are limited, increase access to basic services in underserved and vulnerable communities, and allow for task shifting to alleviate overstretched health systems (Perry et al. 2014). In their role supporting HIV patients in sub-Saharan Africa, CHWs reduced staff workload and patient wait times at healthcare facilities, expanded reach of health services in communities, increased uptake and quality of HIV services, and improved patient retention in care (Mwai et al. 2013). CHWs can also play a beneficial role in improving health outcomes and reducing community burden of NCDs, while improving equity in health care delivery and service (Jeet et al. 2017)]. A systematic review by Kim et al. (2016) reported positive outcomes for CHW interventions and cardiovascular disease risk reduction, as well as blood pressure and type 2 diabetes control. Previous work also demonstrates the potential of CHW-led interventions in increasing physical activity levels (Costa et al. 2015).

Following international guidelines for the expansion of activities performed by CHWs (Singh and Sachs 2013), the role of the CHW in primary care has expanded greatly over the last decade. In 2010, the South African National Department of Health launched a national primary health care initiative, which called upon CHWs to serve on ward-based primary health outreach teams providing education, promoting healthy behaviours, and supporting community member linkage to health services and health facilities (Mhlongo and Lutge 2019). This has led to CHWs providing greater levels of health and social services to community members for a range of conditions, including the screening, referral, and management of NCDs, such as type 2 diabetes and hypertension (le Roux et al. 2015; Morris-Paxton et al. 2018; Ramukumba 2020). Another unique CHW role is the ability to bring healthcare services to patients in their own homes. CHW-led home visits have proven beneficial for multiple health-related activities including immunization efforts, healthy development of children, and maternal health (Tripathi et al. 2016; Stansert Katzen et al. 2021).

Despite these advances, little is known about the role of CHWs in overcoming barriers and expanding the reach of NCD prevention efforts, particularly in historically disadvantaged communities. Outside of a few efforts in North America (Vidoni et al. 2019), efforts to investigate CHW-led, home-based NCD prevention and management activities are relatively sparse. Therefore, the goal of this study was to examine the feasibility and acceptability of CHW-led home visits for NCD risk reduction in a low-resource community.

## Methods

### Study overview

This study took place in conjunction with a series of home visits by CHWs to conduct health screening assessments in Soweto, South Africa in September 2021. The home visits consisted of: 1) CHWs greeting residents, introducing themselves, and explaining the purpose of their visit, 2) obtaining permission to enter the home, 3) performing the informed consent process, 4) conducting a standardised health assessment protocol, and 5) completing a brief satisfaction survey at the end of the visit. Residents who gave consent to be recontacted for a follow-up interview were then contacted within 3 days to participate in a semi-structured interview to gain a deeper understanding of their experience with the CHW-led health assessment conducted in their home setting.

### Description of community health workers (CHWs)

The CHWs were young adults (age 18–30 years) undertaking training in health promotion, health behaviour change support, and basic community health screening as part of an accredited community health work qualification (South African National Qualification Framework Health Promotion Officer/Community Health Worker). Standard minimum entry requirements for community health work applied. The training programme formed part of a youth employment initiative and research programme operated by the Wits Health Hub (www.witshealthhubb.org) with funding support from government. Therefore, youth were eligible for this program only if they were from the local community and not already in employment, education, or training. Selection for the programme involved basic language and mathematics competency tests and interviews to assess core competencies for the role.

### Study design and setting

The home-based health screening was conducted in Soweto within a 5-km radius of the CHW training facility. The training facility, focused on youth development, is in an area of historical deprivation characterized by high-density housing (hostels and flats) that has been targeted in recent years for economic urban development and infrastructure investment. In 2021 alone, there were 398 reported assaults, 50 murders, 20 attempted murders, 92 sexual crimes, and 1,137 total contact crimes reported for this area (Institute for Security Studies issafrica.org/crimehub 2022). Twenty CHWs went in pairs door-to-door in the selected community during September 2021 to conduct health screening assessments, which are 20–35 minutes in duration. During the visit, household members received details of the study and were assessed for their eligibility to participate. Eligibility criteria included being: 1) at least 18 years old, 2) willing to provide written informed consent, 3) fluent in English, and 4) not displaying any symptoms of COVID-19 (determined by an infrared, non-contact thermometer reading of ≤ 37.5°C or the presence of symptoms). Before proceeding with any study procedures, household members were asked to provide their written informed consent, as well as consent to be contacted for a follow-up interview.

### Procedures for home visit

Eligible household members that provided informed consent had their height and waist circumference measured to the nearest 0.1cm using a disposable tape measure following standardized World Health Organization measurement protocols (de Onis et al. 2004). Thereafter, seated brachial blood pressure was measured using an automated device (M10-IT; Omron Healthcare, Kyto, Japan) following International Society of Hypertension (ISH) guidelines (Unger et al. 2020). Participants with a blood pressure of 140/90 mmHg or above were provided with a referral to the local clinic for follow-up. The participants were then asked to complete a health questionnaire to obtain self-reported medical history (known hypertension, current hypertension treatment, known type 2 diabetes mellitus, previous heart attack/stroke, past COVID-19 infection, COVID-19 vaccination status) and health behaviour (tobacco and alcohol usage). Thereafter, physical activity was assessed using the Physical Activity Vital Sign (PAVS) questionnaire (Sallis et al. 2016; Stoutenberg et al. 2017) asking participants, on average, how many days per week they engage in moderate to strenuous exercise and for how long.

### Satisfaction survey

Following the health screening, the household members were asked to compete a short satisfaction survey regarding their experiences with the CHWs and the home visit. The satisfaction survey queried them on their general attitudes towards receiving home-based screening and guidance from the CHWs, their perceived monetary value of the visit, and how they felt about their health [using a scale from 0 (worst) to 100 (excellent)] prior to and after the visit from the CHW. Satisfaction surveys were administered through the tablets and completed in English. Study data were collected and managed using REDCap electronic data capture tool (Harris et al. 2009) hosted at the University of the Witwatersrand.

### In-depth interviews

After completing the measurements and the satisfaction survey, participants were asked if they would be interested in participating in an in-depth interview in the future to share their general attitudes towards the home health visit, the blood pressure and physical activity screening, and the guidance received from the CHWs. The in-depth interviews were conducted within 3 days of the initial home visit to promote adequate recall. All interviews were recorded and transcribed verbatim. Interviews were conducted in English when possible, or in the participant’s preferred language with transcription and translation of audio recordings by transcribers checked by the CHW and the research team.

### Data analysis

#### Health data and satisfaction surveys

Central obesity was categorized as a ratio of waist circumference to height of ≥ 0.5 (Ashwell and Gibson 2014). Hypertension was defined as a blood pressure ≥ 140 mmHg systolic or ≥ 90 mmHg diastolic or currently taking anti-hypertensive medication (Unger et al. 2020). For those not on anti-hypertensive medication, elevated blood pressure (prehypertension) was defined as 130–139 mmHg systolic or 85–89 mmHg diastolic, following the Hypertension Practice Guidelines (Unger et al. 2020). For assessments captured as continuous variables (i.e., age, years of education, height, waist circumference, systolic and diastolic blood pressure), visual inspection of histograms informed the normality of data, and the mean and standard deviation were reported for normally distributed data. Median and interquartile ranges were reported for non-normally distributed data, while absolute numbers and percentages were reported for categorical variables. To test for differences between men and women in the sample, an independent *t*-test, Mann–Whitney U test, or chi-square test was used. The Wilcoxon signed-rank test was used to compare repeated measures within the same sample. Statistical data analysis was conducted in SPSS 24.0.

#### Follow up interviews

Interview transcripts were analysed using thematic analysis, as outlined by Braun and Clarke (2006). A combination of deductive and inductive analyses was used to assess participant experiences engaging with the CHW home visits. Two research team members (the raters) read a subset of transcripts to develop an initial codebook based on participant responses. The raters (LM & AL) coded two transcripts together to refine the codebook. The research team met regularly to discuss codebook changes, verify that codes were applied systematically, and reach consensus on discrepant ratings. Five transcripts were selected to assess the percentage of agreement between raters, calculated by the number of times both raters assigned the same code to a text segment. Initially, the two raters agreed on 96.4% of the independently coded data, but 100% consensus was reached through further discussion. Coded text was entered into Dedoose (version 7.0.23) to perform the content analysis and to extract coded participant responses. Codes were sorted into categories derived from the interview guide. The research team reviewed all codes and categories to identify meaningful themes.

### Ethics

Ethical approval was grant by the Human Research Ethics Committee (Medical) at the University of Witwatersrand [Ref. M200941 and M170334] for all study materials and procedures.

## Results

### Study sample

**Fig. 1:**
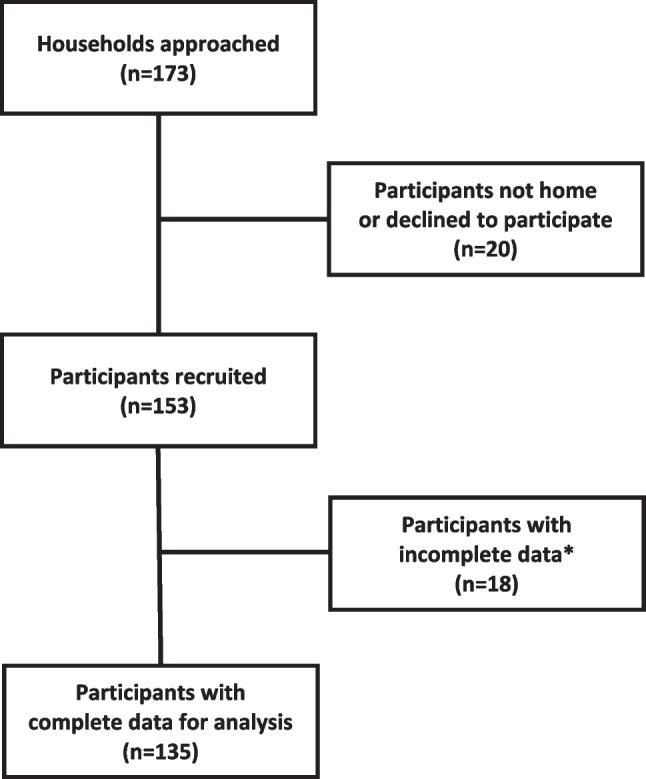
Study flow diagram. * Participants were unable to complete the measurements and/or questionnaire due to previous scheduled appointments or voluntary withdrawal

The CHWs visited 173 community households during the study period (Fig. 1). Individuals at three homes (2%) did not come to the door and a further nine individuals (5%) were not interested in participating. Of the remaining 161 community members, 153 (95%) gave their consent to take part in the study. Eighteen participants were either unable to complete (due to time commitments) or withdrew during the assessments. A final sample of 135 participants (56 men, 79 women), ranging from 18–83 years of age (median age: 38 years) agreed to participate (Table 1). More than half of the participants reported having 7–12 years of education, with approximately one third reporting > 12 years of education. The prevalence of central obesity was higher in women than men (81% vs 36%, *p* < 0.001). One in five individuals reported never having checked their blood pressure previously. Based on the readings taken during the home visit, 36% of the participants were identified as having hypertension, compared to 11% of participants who reported knowing they had hypertension. There were significantly more male smokers compared to female smokers (55% vs 19%, *p* < 0.001), while 21% of participants reported regularly consuming alcohol (daily or weekly). More than half of the sample (56%) reported not achieving 150 minutes per week of physical activity.

**Table 1:** Distribution and characteristics of the study population

Characteristic	Total sample (*n* = 135)	Males (*n* = 56)	Females (*n* = 79)	*P*-value
Socio-demographic				
Age, years (median, IQR)	38 (29)	35 (29.5)	39 (28)	0.345
Number years education				
No education: *n* (%)	6 (4.4)	2 (3.6)	4 (5.1)	0.087
1-6 years: *n* (%)	6 (4.4)	5 (8.9)	1 (1.3)	
7-12 years: *n* (%)	77 (57.0)	27 (48.2)	50 (63.3)	
> 12 years: *n* (%)	46 (34.1)	22 (39.3)	24 (30.4)	
Anthropometry				
Height, cm	164.7 ± 9.6	170.6 ± 7.3	160.5 ± 8.8	< 0.001*
Waist circumference, cm	91.0 ± 18.0	86.1 ± 19.9	94.4 ± 15.8	0.008*
Waist-to-height-ratio	0.56 ± 0.12	0.50 ± 0.11	0.59 ± 0.11	< 0.001*
Central obesity (WHtR > 0.5)^#^: *n* (%)	84 (62.2)	20 (35.7)	64 (81.0)	< 0.001*
Systolic blood pressure, mmHg	124 ± 16	123 ± 13	124 ± 18	0.711
Diastolic blood pressure, mmHg	84 ± 10	82 ± 10	85 ± 11	0.078
Hypertension status: *n* (%)	39 (32.0)	12 (27.9)	27 (34.2)	0.198
Normotensive	66 (48.9)	29 (51.8)	37 (46.8)	0.198
Elevated blood pressure	20 (14.8)	11 (19.6)	9 (11.4)	
Hypertensive	49 (36.3)	16 (28.6)	33 (41.8)	
Self-reported medical history				
Hypertension prior diagnosis*: *n* (%)	15 (11.1)	5 (7.1)	11 (13.9)	0.217
Diabetes mellitus: *n* (%)	5 (3.9)	0 (0)	5 (6.3)	-
Previous heart attack: *n* (%)	2 (1.5)	2 (3.6)	0 (0)	-
Previous stroke: *n* (%)	2 (1.5)	1 (1.8)	1 (1.3)	-
Previous COVID-19 positive: *n* (%)	6 (4.5)	3 (5.5)	3 (5.5)	0.66
COVID-19 vaccinated: *n* (%)	42 (31.1)	14 (25.0)	28 (35.4)	0.258
Behavioural health factors				
Tobacco use: *n* (%)				
Current use	46 (34.1)	31 (55.4)	15 (19.0)	< 0.001*
Past use	21 (15.6)	8 (14.3)	13 (16.5)	
Never used	68 (50.4)	17 (30.4)	51 (64.6)	
Alcohol consumption: *n* (%)				
Daily	1 (0.7)	1 (1.8)	0	0.076
1-6 times per week	27 (20.1)	15 (26.8)	12 (15.4)	
1-3 times per month	50 (37.3)	23 (41.1)	27 (34.6)	
Never/rarely	56 (41.8)	17 (30.4)	39 (50.0)	
Physical activity less than 150 min/week: *n* (%)	75 (55.6)	26 (46.4)	49 (62.0)	0.072
Last BP check: *n* (%)				
Never	29 (21.5)	19 (33.9)	10 (12.7)	0.007
Over 12 months ago	30 (22.2)	13 (23.2)	17 (21.5)	
Within the last 12 months	76 (56.3)	24 (42.9)	52 (65.8)	

*BP*, blood pressure; cm: centimeter; *IQR*, interquartile range; *WHtR*, waist-to-hip ration

* = statistically significant

### Post-home visit satisfaction survey

At the conclusion of the home testing, participants were asked if CHWs had visited their home previously to conduct similar health checks. Of the 135 participants, 130 (96%) reported not receiving a similar home health check in the past. When asked about their experience with the home visit, 97% of participants reported that it was ‘very easy’ or ‘easy’ to understand the information during the visit, 91% reported that the CHWs seemed very knowledgeable, and 100% reported that the CHWs answered their questions ‘very well’ or ‘well”. Additionally, 93% of the participants reported that they would be ‘very likely’ or ‘likely’ to request this home service again if it were available. Participants reported feeling average (median score = 50) about their health prior to the visit, which significantly increased to 88 by the end of the visit (*p* < 0.001). Participants estimated the value of the home visit at 68 Rands (IQR 42 Rands), which was equivalent to approximately $4.30 USD at the time of this study.

### Follow up individual interviews with community members

Seventy-nine community members (27 men, 52 women) agreed to be contacted again for follow-up interviews, with 62% expressing a preference for in-person rather than telephone interviews. Upon recontact, only 29 participants were accessible for the interviews. One recording was inaudible, resulting in a total of 28 individual interviews (22 in-person, six by telephone) for analysis (five men, 23 women). The median age of the individuals interviewed was 39 years (range: 18–76 years) with 21% (*n* = 6) having > 12 years of education, 64% (*n* = 18) having 7–12 years of education, and 14% (*n* = 4) having <7 years of education. Nine individuals (32%) reported engaging in 150 minutes or more of moderate to vigorous activity per week, while seven (25%) of individuals were normotensive, seven (25%) were prehypertensive, eight (29%) of individuals had undiagnosed hypertension, and six (21%) individuals were hypertensive, but receiving treatment. Four main themes, described below, were identified from the interview transcripts relating to the CHW engagement with the participants, with sample quotes presented in Table 2.

**Table 2:** Sample of quotes organized by theme

Theme	Sub-themes	Quotes
Receptivity to home visits from health advocates	First impressions	“…so I thought maybe it is regarding COVID. But then they made it clear that they here for high blood [colloquial term for hypertension] and continued to say they here to see what really troubles me.” “The way they were talking to me, it was very respective and they were just open in general. They were just talking to me and they made me very comfortable. That’s why I allowed them to come in.” “Yes, when I looked at him, I saw a trustworthy person.” “Oh, when they mentioned that they will be checking my high blood, you see, because it’s not something that you check as often. And there’s too many diseases now. (I: That’s right.). That’s the reason I decided to let them in.”
	Accessibility	“What I’ve learnt or what I’ve loved is getting visitors from people who are from the health, if this can continue to other communities it could help a lot of people, especially those who can’t go by themselves to the clinics, if maybe healthcare workers can go around checking people, like high blood, sugar diabetes all those things.” “It makes me very happy, I feel very glad, I truly wish that you would check on us every second day…” “I think they should come more often and do other things so we don’t go to the clinic because it’s always full…”
Receptivity to advice received	Empowerment from advice	“I feel good because most of the time at the clinic they just pump us and they don’t explain anything (I: Ohhh…) so now I got to understand what it means when its like this and when its like that.”
	Recollection of advice received	“He asked me to reduce significantly the amount of fats I consume, not that I should not eat fats at all, but I should try introducing vegetables to my diet, I should sometimes just try to buy and eat vegetables like potatoes, cabbage and spinach especially for dinner without meat or pap. And eat a lot of fruits as well. He told me that he isn’t saying I’m sick.” “I learnt that a person should always take care of themselves, and that life is too short. We must always check what’s happening in our bodies so that you can identify things that are not ok so that you can be able to fix it while you still have time.”
	Acting on advice	“I’ll start going to clinic, start taking my high blood treatment. I was lazy, but now I will go and take the pills if I have.”
Experience with blood pressure screening	Experience	“She said she’s not supposed to take my BP while I’m standing, I should be seated. Then I sat, and she then sanitized the cuff, then she sanitized me then herself. Then she put the cuff on me and then she started taking my BP.” “They pulled out the machine to check my blood pressure, and their finding was that it is high.” “He said I must just relax, not panic, put my hand straight and calm down because there’s nothing hard I must just calm down and just relax.” “What I remember is that they stated that I mustn’t put my legs together and I must not panic. So that the numbers are accurate, because some of the time when you panic, it goes up and it would be like I am sick, while in am not sick.” “No, they did a good job; there is nothing I can say needs to be added because everything went well.”
	Comprehension	“It was easy because of they explain, they explain very well, that even if you don’t understand you can ask question.” “Yes, I understood well because he explained, he showed me on the machine.” “They explained everything well.”
Receptivity to physical activity advice	Physical activity advice received	“They said I must exercise, then I said to them but as I’m cleaning doing the house chore I’m exercising and they laughed, they said I should gym and stretch my legs and I told them okay I will do them in my bedroom.”
	Preferences for exercise modalities	“Just walking maybe from normal walking to brisk walking, and just exercises that feel like you are opening overhead cupboards over and over, you see, I wouldn’t like jumping up and down because I’m not young like you anymore.” “Just exercises so I lose weight, stomach exercise you see and get a diet on what to eat so my body will be fine and not get sick.”
	Barriers and facilitators to referral to a community center	“The only thing that would stop me would definitely be when I am not feeling well, or these boys are not there *demonstrating* (money I guess). Because we use that to survive.” “Yes, I would like to join but now the problem is there’s a joining fee and when you not working it’s hard to join things like that, like me where would I get the money?” “It is a good thing; it will be within reach for us; the community and exercise is part of health; we have to exercise and minimize our visits to the clinics saying, ‘my BP is high’ or ‘my sugar levels are high.’”

#### Receptiveness to home visits

In most cases, community members spoke about the need to go to local health clinics for check-ups, medication, and illness evaluation. For these people, having healthcare workers come to their door was unusual. Their initial thought in seeing the professionally dressed CHWs with uniforms and nametags were that they were ‘knocking on their front door’ for something pertaining to the COVID-19 pandemic and vaccine administration. Once the CHWs explained the purpose of their visit, community members expressed feeling safe and comfortable letting them into their homes. Community members described the CHWs as respectful and knowledgeable and expressed how much they appreciated the home visit, stating that they were very satisfied with their visit and learned a lot from the CHWs. Many conveyed the difficulties of visiting the local clinic including the long wait times, unfriendly staff, and a lack of explanation of medical issues. One interviewee commented that people will be very sick, “but they are too scared to go to the clinic.” The community members were grateful that people cared for their health, and wished the home health checks could be offered more frequently and to everyone in the ‘hood’. One interviewee shared, “I was so happy, because it shows that we also matter”, demonstrating the rarity of the home visits and the need for more accessible health care.

#### Receptivity to advice received

Community members expressed an appreciation for receiving health advice from the CHWs. They were given the opportunity to learn how to measure their own blood pressure, advised on their dietary intake of salt and fat, and consulted on their general physical activity. Those interviewed described generally feeling empowered by the home service and care. There was no negative feedback from the community members regarding the advice they received. Overall, community members described feeling motivated to follow the CHW’s advice: “I’ll start going to clinic, start taking my high blood treatment. I was lazy, but now I will go and take the pills if I have.”

#### Experience with blood pressure screening

While recalling their experience with the home visit, community members remembered being told by the CHWs to remain calm, relaxed and still so that they could get an accurate measurement of their blood pressure. Some community members also recalled the CHWs telling them that their blood pressure was elevated over the normal value, leading to an increased awareness of their health status. Overall, community members expressed satisfaction with the experience of having their blood pressure measured and there was little feedback as to how the process could be improved. Community members also reported they had no issues comprehending what the CHWs were doing, asking, and explaining. Community members reported easy, clear communication, being able to ask questions, and receiving thoughtful, understandable responses.

#### Receptivity to physical activity advice

Those interviewed were receptive to the advice they received regarding their physical activity level. Older participants expressed a preference for engaging in lower intensity activities that fit within their daily routines, such as walking and stretching, while younger participants voiced a desire for exercising to lose weight. Several women discussed finances and childcare as barriers. The interviewees were receptive to the idea of receiving referrals to join exercise programs at a local community centre, and commented on the presence of a local exercise/training facility as a positive addition to their community: “It is a good thing; it will be within reach for us; the community and exercise is part of health; we have to exercise and minimize our visits to the clinics saying, ‘my blood pressure is high’ or ‘my sugar levels are high.’”

## Discussion

Innovative strategies are needed to address NCDs in LMICs. One emerging global strategy is using CHWs to reach vulnerable populations in under-resourced settings (Khetan et al. 2018; Long et al. 2018; Rawal et al. 2020; Bysted et al. 2022). Reviews have found that over 60% of CHWs in urban settings performed some sort of home-based care; however, most of these efforts focused on HIV/AIDS, maternal, new born and child health, geriatric care, mental health, substance abuse, and immunization administration (Perry et al. 2014; Ludwick et al. 2020). Expanding on this previous work, our study investigated the feasibility and acceptability of CHWs conducting standardized, home-based health screening assessments and brief counselling for blood pressure and physical activity.

Overall, our home-visit approach proved to be highly feasible in a low-resource community with > 95% of households consenting to participate in the health screenings. This level of participation is similar to a home-based screening program in rural India in which nearly 90% of targeted households were covered (Basu et al. 2019). We also found high levels of acceptance towards the home visits, responsiveness to the advice provided, and a willingness to learn about their cardiovascular health. This is both a milestone for NCD implementation research and an important finding in a community where typically only one in every five individuals has sufficient health literacy (Calvert et al. 2022).

Household members expressed a high level of comfort with the CHW-led home visits, a surprising finding given the high levels of crime reported in the area, but similar to previous work conducted in South African settings (Medina-Marino et al. 2021; Ngcobo and Rossouw 2022), suggesting the trusted role that CHWs occupy in the community. Home-based tuberculosis testing, lasting as long as 2 hours, was acceptable due to its convenience (i.e., not having to make multiple trips to the health clinic) and receiving reliable information on the spot (Medina-Marino et al. 2021). In another study, community members expressed greater receptivity for the home visits when informed of the role, age, and gender of the CHWs prior to the visit (Ngcobo and Rossouw 2022). Our finding is particularly meaningful given that the home visits were conducted during the height of the COVID-19 pandemic, amidst societal lockdowns and physical distancing. Despite these barriers, household members welcomed CHWs into their homes. This may be due to the high level of professionalism displayed by the CHWs, who had recently completed rigorous training that provided them with the knowledge and skills to give accurate and meaningful advice, as well as their use of uniforms and nametags.

Follow-up interviews revealed high levels of community member satisfaction with the individualized care that they received, something many remarked that they were not receiving at the local health clinic. While previous work has found high levels of patient satisfaction with healthcare services in South Africa (Jacobsen and Hasumi 2014) and in local health clinics in the same region as the current study (Nunu and Munyewende 2017), our study participants expressed that clinics were very busy, forcing staff to move quickly between patients, limiting individual patient interactions. This is similar to other work that found the most common problems experienced with healthcare services in South Africa included long wait times, unfriendly staff, and being turned away from clinics (Hasumi and Jacobsen 2014). Home visits may alleviate concerns about going to health clinics, while providing high quality, accessible, and individualized care in communities facing numerous health issues and a lack of resources.

Community members in the current study expressed appreciation for the individualized care that they received, including an explanation of their blood pressure reading and receiving advice on improving their overall cardiovascular health. CHWs are effective in providing community members with hypertension guidance and helping residents improve chronic disease conditions (Kangovi et al. 2017). Further, home-based blood pressure screening allows for identifying potential blood pressure problems and achieving population-level blood pressure improvements in South Africa (Sudharsanan et al. 2020). A home-based blood pressure screening in Kenya proved to be a feasible strategy for screening a broad array of community members for hypertension and diabetes and identifying a large pool of high-risk individuals (Pastakia et al. 2013).

Community members were also highly receptive to the consultation they received regarding their dietary and physical activity habits. While previous work has demonstrated that CHWs are effective in promoting physical activity (Costa et al. 2015), physical activity screening and promotion in the home setting has not been widely examined. One study that utilized CHW home visits for individuals with type 2 diabetes and low incomes in the United States found that physical activity levels and dietary behaviours significantly improved in those randomized to the self-management intervention (Gray et al. 2021). This suggests that behavioural counselling by CHWs may be an effective strategy for improving NCD-related risk facts. Community members were also open to the idea of being connected to community-based physical activity resources. A systematic review found that in 38 of 114 studies involving hard-to-reach populations, CHW outreach efforts included referring and linking community members to health services (Ludwick et al. 2020), although none involved a referral to physical activity programs. Access to facilities that are safe and able to provide space for activities from a trusted source or community member can be a key factor to promote physical activity and health behaviours for members of this community (Ware et al. 2019). Future work should investigate community member referral to local health-promoting resources, such as physical activity and healthy eating programs, and strategies for overcoming noted barriers, such as cost of the programs and childcare resources.

This study has several notable strengths. First, we systematically sampled many households in a predefined geographical area in an under-resourced community. Second, the home-based health assessments were rigorously conducted by trained CHWs supervised by professional staff following a standardized protocol. Finally, we explored community members acceptability of the CHW home visit immediately after through a satisfaction survey, as well as through more in-depth interviews. At the same time, this study has several limitations. First, this work was conducted during the height of the COVID-19 pandemic, leading to the possibility that individual behaviours may have been affected by recent community lockdowns and curfews. The study sample (i.e., 82.1% of follow-up surveys were conducted with women) may not be sufficiently representative to identify sex differences. Further, home visits were conducted during weekdays, which may exclude individuals engaged in full-time employment or academic study. While work-from-home policies were more common during the COVID-19 pandemic, low-income communities were frequently least able to implement this practice (Garrote Sanchez et al. 2021). Finally, since the study engaged CHW trainees, they had a lesser workload than full-time employed CHWs, allowing them more time and care with each household member. However, this research fills an important gap in understanding how to embed CHW programs into a community, identified as a key success factor across a range of health service provision in multiple countries and across the lifecourse (Scott et al. 2018).

### Conclusion

Although there have been ongoing efforts to improve primary care in South Africa, progress is often tempered by challenges, such as resource distribution, management, and leadership struggles (Maphumulo and Bhengu 2019). The success of the home visits in this study highlights the potential to expand accessible, community-based health care opportunities for NCD prevention and management, as the blood pressure and physical activity screening and brief counselling were well-received and understood by the community members. With the continued expansion of CHW roles and responsibilities assigned through the task shifting of health-care services in under-resourced communities, CHWs in LMICs may be the critical force needed to address the growing tide of NCDs through primary prevention and health promotion.

## Data Availability

The data underlying this article will be shared on reasonable request to the corresponding author.
